# circACTR2 attenuates gemcitabine chemoresiatance in pancreatic cancer through PTEN mediated PI3K/AKT signaling pathway

**DOI:** 10.1186/s13062-023-00368-8

**Published:** 2023-03-30

**Authors:** Chao Xu, Qinwen Ye, Chao Ye, Shaojun Liu

**Affiliations:** 1 Department of Gastroenterology, Division of Life Sciences and Medicine, The First Affiliated Hospital of USTC, University of Science and Technology of China, Hefei, 230001 Anhui P.R. China; 2 Department of Gastrointestinal surgery, Division of Life Sciences and Medicine, The First Affiliated Hospital of USTC, University of Science and Technology of China, Hefei, China

**Keywords:** Pancreatic cancer, CircACTR2, Chemoresistance, PTEN, AKT signaling pathway

## Abstract

**Background:**

Recently, accumulating studies have unveiled that circRNAs exert critical function in a variety of tumor biological processes including chemoresistance. Our previous study has found circACTR2 is significantly down-regulated in acquired gemcitabine (GEM)- resistant pancreatic cancer (PC) cells, which has not been well-explored. Our study aimed to research the function and molecular mechanism of circACTR2 in PC chemoresistance.

**Methods:**

qRT-PCR and western blot analysis was performed to detect gene expression. The effect of circACTR2 on PC GEM resistance were examined by CCK-8 and flow cytometry assays. Whether circACTR2 could sponge miR-221-3p and regulate PTEN expression were determined by bioinformatics analysis, RNA pull-down, and Dual-luciferase reporter assay.

**Results:**

circACTR2 was notably down-regulated in a panel of GEM-resistant PC cells lines, and negatively associated with aggressive phenotype and poor prognosis of PC. circACTR2 downregulation contributed to GEM chemoresistance of PC cells with decreased S phase ratio of cell cycle and cell apoptosis, as confirmed by gain- and loss-of-function assays in vitro. In addition, circACTR2 overexpression retarded GEM resistance in vivo. Further, circACTR2 acted as a ceRNA against miR-221-3p, which directly targeted PTEN. The mechanistic studies revealed that loss of circACTR2 promoted GEM resistance in PC through activating the PI3K/AKT signaling pathway by downregulating PTEN expression in a miR-221-3p dependent manner.

**Conclusions:**

circACTR2 reversed the chemoresistance of PC cells to GEM through inhibiting PI3K/AKT signaling pathway by sponging miR-221-3p and upregulating PTEN expression.

**Supplementary Information:**

The online version contains supplementary material available at 10.1186/s13062-023-00368-8.

## Introduction

Pancreatic cancer (PC) is one of the most lethal human malignancies, with an overall five-year survival rate of less than 5% [1]. The high mortality of PC could be largely attributive to its highly aggressive nature, wherein local invasion and remote metastasis may occur during the early stages of carcinogenesis, and its inherent chemoresistance [2]. Thus, most patients diagnosed with PC are not operable and chemotherapy is thus the main treatment option. At present, gemcitabine (GEM) is the first-line drug used in the treatment of PC. However, its therapeutic efficacy is far from satisfactory due to the inherent chemoresistance of PC [3]. A previous study revealed that only 23.8% of GEM-treated patients received therapeutic benefits in their early stages of treatment [4]. What’s worse, acquired GEM resistance makes PC treatment more difficult. The molecular mechanisms of GEM resistance in PC include aberrant gene expression, mutations, and deregulation of key signaling pathways such as nuclear factor κB (NF-κB), Akt and Notch pathways [5]. However, a better understanding of the molecular mechanisms underlying the development of GEM chemoresistance is still necessary to develop novel-targeted therapies to ‘flip the switch’ from drug resistance to susceptibility in PC.

Circular RNA (circRNA) is a special class of non-coding RNA molecules, characterized by covalently closed circular structures through special selective splicing. Due to the lack of 3 ‘end and 5’ end, circRNA is not easy to be degraded by exonuclease and is more stable than linear RNA. Accumulating studies have shown that circRNA can regulate gene expression at transcription and post-transcription level by cis-regulation of transcription, alternative splicing of RNAs, encoding peptides, and acting as competing endogenous RNAs [6]. In addition, circRNA has been found to play an important role in tumorigenesis and tumor progression by modulating tumor cell proliferation, differentiation, apoptosis, invasion, and drug resistance [7, 8]. Recently, several circRNAs have been discovered to participate in chemoresistance of PC. For example, hypoxic exosomal HIF-1α-stabilizing circZNF91 promotes chemoresistance of normoxic PC cells via enhancing glycolysis [9]. Another study reported that circLMTK2 knockdown attenuated GEM resistance of PC cells by regulating PAK1 via miR-485-5p [10]. Our previous study has revealed the functional profiles of differentially expressed circRNAs associated with GEM resistance in PC cells [11]. However, the biological functions and potential mechanisms of these dysregulated circRNAs associated with PC chemoresistance remain unclear, thus need to be clarified to to make a breakthrough in the clinical application for PC treatment.

In the present study, we found that circRNA_102747 (also named circACTR2) was notably downregulated in GEM-resistant PC cells, and was necessary for the maintenance of GEM resistance. By acting as a ceRNA by sponging miR-221-3p, circACTR2 upregulated PTEN expression and consequently inhibited PI3K/AKT signal pathway to attenuate GEM resistance of PC. Our study provides a novel therapeutic target for overcoming PC GEM cheomresistance.

## Materials and methods

### Cell culture and transfection

Human PC cell lines (PANC-1, SW1990, BxPC-3, CFPAC-1 and AsPC-1) were purchased from the Type Culture Collection of the Chinese Academy of Sciences (Shanghai, China). SW1990/GZ cells are acquired GEM-resistant SW1990 cells which were established in our lab [11]. PC cells were cultured in DMEM (Thermo Fisher Scientific, Waltham, MA, USA) supplemented with 10% fetal bovine serum (Thermo Fisher Scientific), 100 U/mL penicillin, and 100 µg/mL streptomycin. Cultures were maintained at 37 °C in a humidified atmosphere of 5% CO_2_.

To knockdown and overexpress circACTR2, lentiviral vector (GenePharma Corp, shanghai, China) was used to express shRNA specifically targeting the junction region of the circACTR2 sequence (AAACTTTCTGATCTTATG) and full length of circACTR2, respectively. Only the empty lentiviral vector was used as a negative control (NC). The mimic and inhibitor of miRNA-221-3p, Phosphatase and tensin homolog (PTEN) overexpression vector and sh-PTEN vector were synthesized by GenePharma (Shanghai, China). For stable transfection, cells were seeded in a 24-well plate until 90% confluent, then transfected with vector using lipofectamine™ 2000 reagent (Invitrogen, Carlsbad, USA) according to the manufacturer’s instructions.

### Clinical specimens

In total, 25 PC samples were collected from the First Affiliated Hospital of USTC between 2018 and 2020. All samples were immediately frozen in liquid nitrogen after resection and stored at − 80 °C for RNA extraction. We have obtained informed consent from all patients and this study was approved by the Ethics Committee of the First Affiliated Hospital of USTC (Hefei, China).

### qRT-PCR

Total RNA was extracted from cells and tissues using TRIzol Reagent (Invitrogen). cDNA was then synthesized using 1 µg samples of the total RNA with SuperScript III Reverse Transcriptase (invitrogen). Real time-PCR was performed using a SYBR primescript qRT-PCR kit (TaKaRa, Japan) on the Step One platform (Applied Biosystems, Shanghai, China). Primer sequences for candidate genes were listed in Additional file 2: Table S1. GAPDH was used as internal reference for quantification of circRNA and mRNA, while U6 for miRNA. Relative expression levels of genes was calculated by 2^–ΔΔCt^ values.

### Western blot assay

The total protein lysate extracted from the PC cells was separated by SDS-PAGE and transferred to PVDF membranes. After blocking membranes, PVDF were incubated with appropriate dilutions of specific primary antibodies against PTEN, GAPDH (Abcam, Cambridge, MA) and Akt, p-Akt (Ser 473) (Cell Signaling, Danvers, MA). For protein visualization, the blots were incubated with HRP-conjugated secondary antibodies after extensive washing with TBS and then visualized using the ECL system.

### Cell viability assay

The viability of PC cells was determined by Cell Counting Kit 8 (Dojindo, Japan) and measured at OD 450 nm with the BioTek Gen5 system (BioTeck, USA). 96-well plates in triplicates at a density of 1.5 × 10^3^ cells per well. The cells were then grow in complete media for another 72 h with indicated concentration of GEM treatment.

### Apoptosis assay

FITC Annexin V cell apoptosis detection kit I (BD Biosciences, CA, USA) was used for cell apoptosis evaluation. Briefly, Cells were resuspended in binding buffer at a concentration of 1 × 10^6^ cells/mL, then incubated with 0.5 mg/mL Annexin V-FITC and 2 mg/ml PI for 10 min and examined by FACSCalibur flow cytometry (BD Biosciences, CA, USA) to analyze the cell apoptosis.

### Cell cycle assay

Cell cycle kit (BD Biosciences, CA, USA) was used for cell cycle distribution evaluation. Briefly, Cells were collected and pre-cooled 70% ethanol, fixed at 4 °C overnight. Then cells were washed by PBS once and added with 0.5 mL propidium iodide (PI) solution (25 µL 20 × PI and 10 µL 50 × Rnase A), examined by FACSCalibur flow cytometery (BD Biosciences, CA, USA) to analyze the DNA content using the PI channel.

### In vivo assay for drug sensitivity

SW1990/GZ cells transfected with OE-circ and OE-NC were injected subcutaneously into the right flank of 6-week-old female BALB/c mice (Chinese Academy of Sciences, Shanghai, China) (5 × 10^6^ cells in 250 µl of PBS per mouse). Each experimental group included four mice. All mice received an i.p. injection of 50 mg/kg GEM twice a week after tumor formation (tumor size between 100 and 200 mm^3^) for 4 weeks. Animals were monitored daily, and tumor volume was measured every fourth day after treatment. All tumor-bearing mice were euthanized on the 28 th day after treatment [12].

### Fluorescence in situ hybridization (FISH)

The Cy3-labeled probes specific for circACTR and miR-221-3p was synthesized by Genepharma (Shanghai, China). SW1990 cells were hybridized with Cy3-labeled probe overnight and then dyed by DAPI. The signals of the probes were detected by a Fluorescent In Situ Hybridization Kit (Genepharma, Shanghai, China) and observed under a fluorescence microscope (Leica, Wetzlar, Germany).

### Immunohistochemistry (IHC)

The xenograft tumor tissue was fixed with 10% neutral formalin, embedded in paraffin and cut into into 4 μm thick sections. Immunohistochemistry was performed to stain PCNA and tunnel using the procedure as previously described [13].

### Tissue microarray (TMA) and in situ hybridization (ISH)

The tissue microarray containing 99 paraffin-embedded PC samples were purchased from Outdo Biotech (Shanghai, China). The probe for circACTR2 containing a biotin label synthesized by Genepharma (Shanghai, China) was used for ISH analysis. Concisely, TMA was dewaxed and rehydrated, digested using proteinase K and hybridized with the specific circACTR2 probe at 4 °C overnight, then incubated with anti-Digoxin-AP (Roche, Basel, Switzerland) at 4 °C overnight. The tissues were stained with NBT/BCIP (Roche, Basel, Switzerland) and quantified.

### RNA pull-down assay

The biotinylated probe specifically bind to the junction area of circACTR2 was synthesized by Tsingke Biotech (Wuhan, China). The probe was incubated with the beads at room temperature for 10 min for immobilization. Then, the biotinylated beads were incubated with SW1990 cell lysate at 4 °C overnight. The biotinylated beads were magnetically separated and washed, then the bound miRNAs in the pull-down materials were extracted using Trizol reagent and analyzed by qRT-PCR assay.

### Dual-luciferase reporter assay

The sequences of circACTR2 and PTEN-3’UTR and their corresponding mutant versions without miR-221-3p binding sites were synthesized and subcloned into luciferase reporter vector psiCHECK2 (Promega, Madison, WI, USA), respectively. All these plasmids were validated by sequencing. The relative luciferase activity was examined by Dual Luciferase Assay Kit (Promega, WI, USA) in accordance with the manufacturer’s protocols.

### Statistical analysis

Differences between various groups were assessed using one-way ANOVA or Student’s test. The Chi-square was used for testing correlations between circACTR2 expression and clinicopathological variables. The survival rates were evaluated by Kaplane-Meier method and tested by log-rank test. The effects of the clinicopathological variables on overall survival of PC patients were determined by univariate and multivariate Cox proportional hazards regression model. Pearson correlation analysis was performed to determine correlations. All statistical analyses were performed using SPSS 19.0 software. P-values less than 0.05 were considered to be statistically significant.

## Results

### circACTR2 is down-regulated in GEM-resistant PC cells

**Fig. 1:**
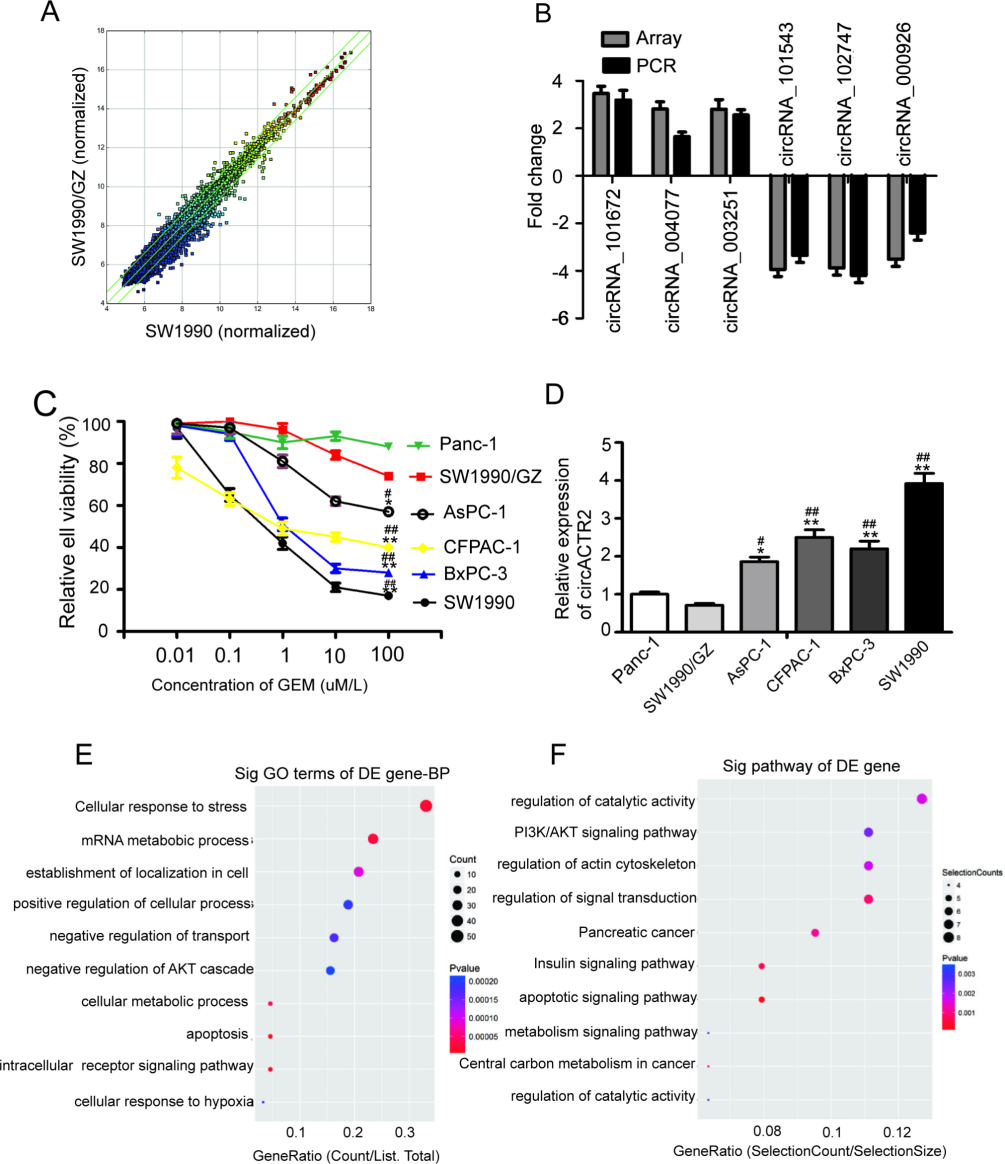
circACTR2 is decreased in GEM-resistant PC cells **A** Scatter plot shows the up-regulated and down-regulated circRNAs in acquired GEM-resistant SW1990/GZ PC cells compared with parental SW1990 cells. **B** The differentially expressed circRNAs with most change fold in SW1990/GZ PC cells compared with SW1990 cells were validated by qRT-PCR. **C** Relative cell viability of a panel of PC cell lines treated with increased concentration of gemcitabine for 72 h was determined by CCK-8 assay. **D** circACTR2 expression was determined in a panel of PC cell lines by qRT-PCR assay. **E** Gene Ontology analysis and **F** pathway analysis of circACTR2 based on microarray profile data. *P < 0.05 and **P < 0.01 vs. SW1990/GZ; ^#^*P* < 0.05 and ^##^*P* < 0.01 vs. Panc-1

Our previous study revealed that 78 circRNAs (fold change ≥ 2) were observed in acquired GEM-resistant SW1990/GZ cells compared with parental SW1990 cells by high throughput circRNA microarray chip (Fig. 1A), and qRT-PCR verification further showed that the expression of circACTR2 has the largest difference with 4.12 times reduction in SW1990/GZ cells among these dysregulated circRNAs (Fig. 1B). We then determined the expression of circACTR2 in a panel of PC cells by qRT-PCR analysis. It showed that the expression level of circACTR2 in GEM-sensitive PC cell lines (IC50 < 10 µM/L GEM) such as SW1990, BxPC-3 and CFPAC-1 is generally higher than that in GEM-resistant PC cell lines (IC50 > 100 µM/L GEM) such as Panc-1 and SW1990/GZ cells (Fig. 1C, D). To determine the potential role of circACTR2 in PC, based on our previous circRNA microarray profile data, Gene Ontology (GO) functional analysis showed that circACTR2 was related to several biological processes related to chemo-resistance, such as cell stress response, cell apoptosis, and intracellular metabolism (Fig. 1E). In addition, pathway analysis showed that it was significantly associated with PI3K/AKT signal pathway (Fig. 1F), which is often abnormally activated to promote chemoresistance of PC. Thus, these results demonstrated that circACTR2 is downregulated in GEM-resistant PC cells, and suggested potential role for circACTR2 in modulating PC GEM resistance.

### circACTR2 is negatively associated with aggressive phenotype and poor prognosis

**Fig. 2:**
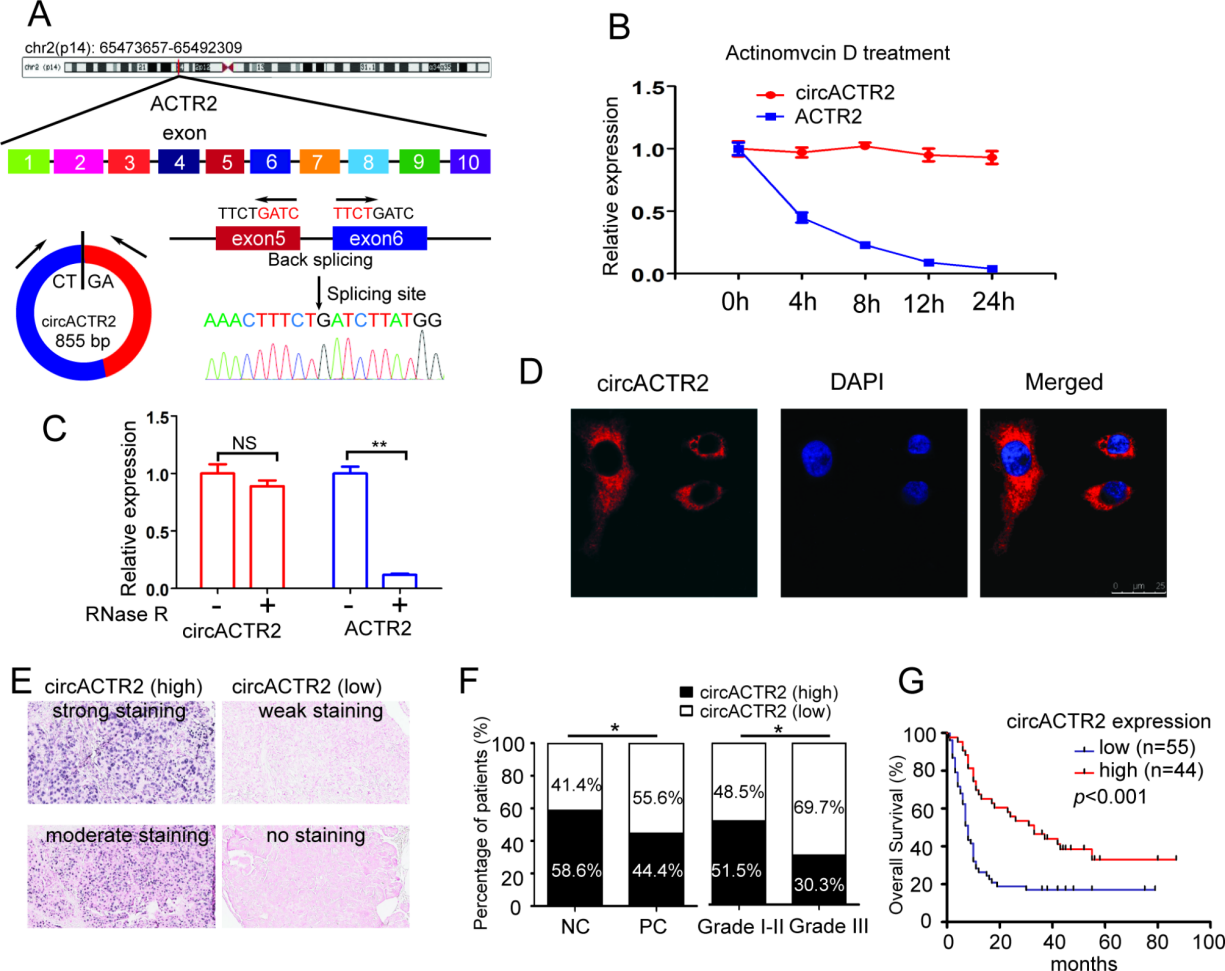
circACTR2 is negatively associated with aggressive cell phenotype and poor prognosis **A** circACTR2 information from the UCSC Genome Browser. The amplified product was sequenced using Sanger sequencing to validate the circularized junction of circACTR2. **B** qRT-PCR analysis of circACTR2 and ACTR2 mRNA in SW1990 cells with actinomycin D treatment for 30 min at 37 °C. **C** qRT-PCR analysis of circACTR2 and ACTR2 mRNA in SW1990 cells treated with RNase R. **D** FISH images showed that circACTR2 labeled with Cy3 (red) was mainly distributed in the cytoplasm of SW1990 cells. The nuclei was stained with DAPI (blue). Scale bar, 25 μm. **E** Representative images of circACTR2 expression in PC tissues were detected by ISH. **F** Percentages of specimen with low or high expression of circACTR2 in PC compared with NP tissues and in PC tissues with different pathological grade. **G** Kaplan-Meier’s analyze of correlation between circACTR2 expression level and overall survival of patients with PC (n = 99, p < 0.01, log-rank test). *P < 0.05; **P < 0.01

According to the UCSC Genome Browser, circACTR2 was spliced from ACTR2 located at chr2:65473657–65,492,309 and finally formed a circular transcript of 855 bp. circACTR2 is generated from back-splicing of the 5th and 6th exons of the ACTR2 gene, the back-spliced regions of circACTR2 was confirmed by Sanger sequencing, and all were in agreement with circBase (Fig. 2A). Resistance to digestion with actinomycin D (Fig. 2B) or RNase R (Fig. 2C) further confirmed this RNA was circular in form. To study the location of circACTR2, hybridization (FISH) assay demonstrated its cytoplasmic localization (Fig. 2D).

We further examined the circACTR2 expression in 99 pairs of PC tissue and adjacent non-cancer (NC) TMA tissue using ISH (Fig. 2E), showing that the expression level of circACTR2 is downregulated in PC tissues compared with NC tissues (Fig. 2F). We then analyzed the correlation between circACTR2 expression and the clinicopathological characteristics as shown in Table 1, demonstrating that circACTR2 expression was significantly correlated with pathological grade with significant downregulation in Grade III compared with Grade I-II (Fig. 2F), while no other significant associations were obtained between circACTR2 expression with gender, age, location of tumor, T stage, N stage, and TNM stage. Additionally, Kaplan-Meier survival analysis showed that patients with low circACTR2 expression had significantly poor overall survival compared with patients with high circACTR2 expression (Fig. 2G). Further univariate and multivariate Cox regression analysis showed that TNM stage, pathological grade and circACTR2 expression levels were independent prognostic factors for PC patients (Table 2).

**Table 1:** The correlation between circACTR2 expression and clinicopathological characteristics of PC patients

Clinicopathological variables	Number of patients in each group	circACTR2 expression		*P* value
		Low (n)	High (n)	
Age (years)				
> 60	48	27	21	0.893
≤ 60	51	28	23	
Gender				
Male	63	36	27	0.674
Female	36	19	17	
Location of tumor				
Head	59	33	26	0.927
Body/tail	40	22	18	
Pathological grade				
І-ІІ	66	32	34	0.045
ІІІ	33	23	10	
T stage				
T1/T2	79	46	33	0.288
T3/T4	20	9	11	
N stage				
N0	56	28	28	0.204
N1	43	27	16	
TNM stage				
І	40	21	19	0.614
ІІ	59	34	25	

* *P* < 0.05

**Table 2:** Univariate and multivariate Cox regression analysis of circACTR2 and survival in patients with PC

Clinicopathological variables	Univariate analysis	*P*	Multivariate analysis	*P*
	HR (95% CI)		HR (95% CI)	
Age (> 60 vs. ≤ 60)	0.785(0.497–1.240)	0.299		
Gender (Male vs. Female)	0.887(0.550–1.432)	0.624		
Tumor location (Head vs. Body/tail)	0.916(0.577–1.454)	0.710		
Pathological grade (І-ІІ vs. III)	0.515(0.321–0.828)	0.006*	0.530(0.326–0.862)	0.010*
T stage (T1/T2 vs. T3/T4)	1.080(0.603–1.935)	0.795		
N stage (N0 vs. N1)	0.576(0.362–0.915)	0.019*		
TNM stage (І vs. II)	0.583(0.360–0.944)	0.028*	0.474(0.287–0.782)	0.003*
circACTR2 (High vs. Low)	0.178(0.105–0.304)	< 0.001*	0.170(0.098–0.296)	< 0.001*

Abbreviations: HR hazard ratio, CI confidence interval

*P < 0.05

### circACTR2 downregulation promotes GEM resistance of PC cells in vitro

**Fig. 3:**
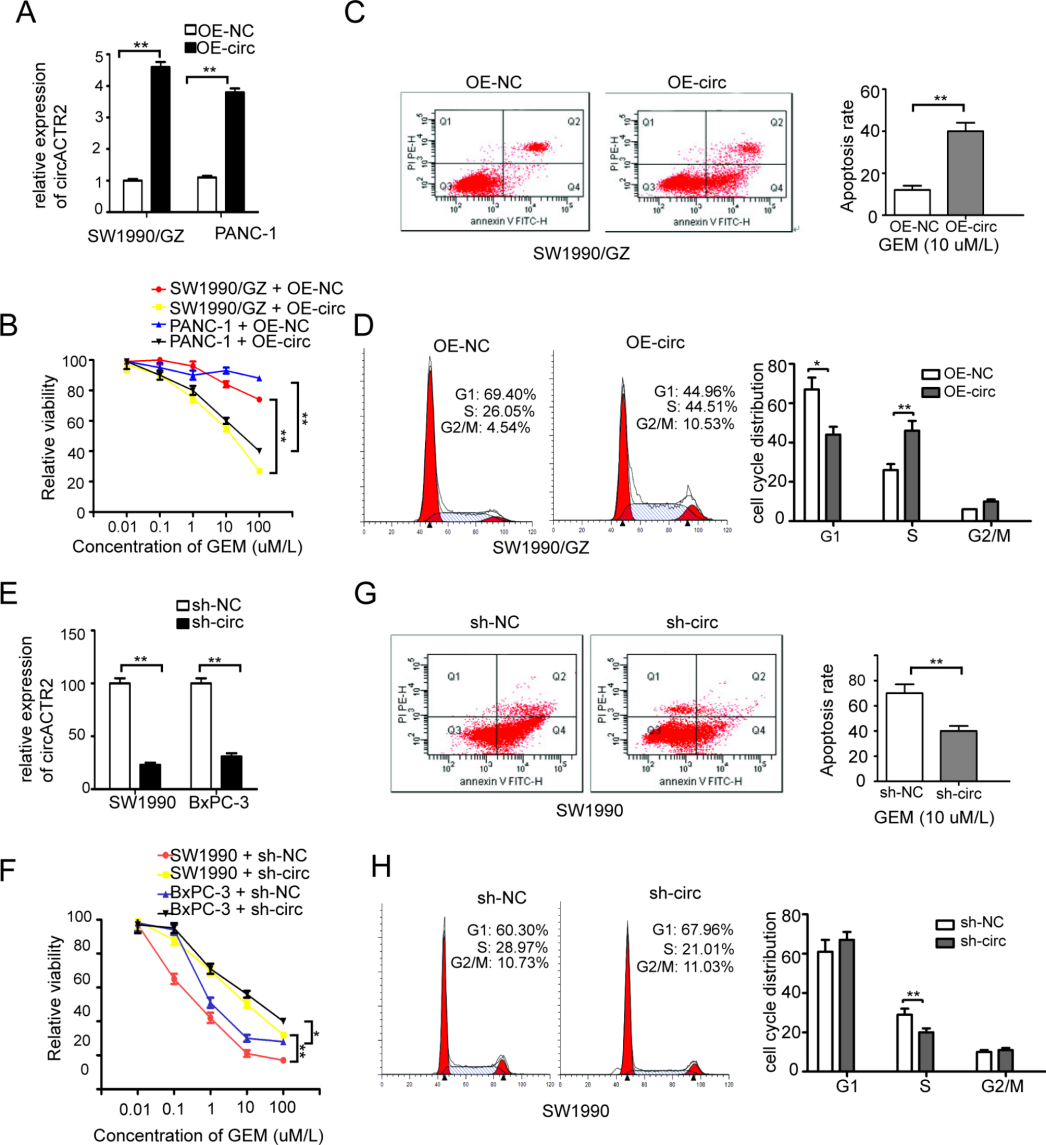
circACTR2 over-expression attenuates GEM-Resistance of PC cells in vitro **A** The relative expression levels of circACTR2 in PC cells stably transfected with circACTR2 over-expression vector (OE-circ) and corresponding negative control (NC) vector were detected by qRT-PCR. **B** Relative cell viability of PC cell transfected with OE-circ vector and OE-NC vector under GEM treatment for 72 h detected by CCK-8 assay. **C** Apoptosis rate and **D** cell cycle distribution in PC cells transfected with OE-circ vector and OE-NC vector detected by flow cytometry. **E** circACTR2 expression in PC cells stably transfected with sh-circACTR2 (sh-circ) vector and sh-NC vector were detected by qRT-PCR. **F** Relative cell viability of PC cell transfected with sh-circ vector and sh-NC vector under GEM treatment for 72 h. **G** Apoptosis rate and **H** cell cycle distribution in PC cells transfected with sh-circ vector and sh-NC vector detected by flow cytometry. *p < 0.05 and **p < 0.01

To determine whether circACTR2 involved in GEM resistance of PC cells, we stably transfected circACTR2 overexpression vector (OE-circ) in SW1990/GZ and PANC-1 cells respectively, we observed a remarkable increase of circACTR2 expression in OE-circ group cells compared with negative control (OE-NC) vector group cells (Fig. 3A). As expected, relative cell viability decreased in OE-circ group cells compared with OE-NC group cells treated with 10 µM/L GEM for 72 h (Fig. 3B). Consistently, the apoptosis rate of OE-circ group cells was increased compared with OE-NC group cells after incubating with gemcitabine (10 µM/L) for 72 h (Fig. 3C). In addition, OE-circ group cells showed reduced G1 phase ratio and increased S phase ratio compared with OE-NC group cells (Fig. 3D). Knockdown of circACTR2 with shRNA (sh-circ) vector transfection led to almost opposite results in SW1990 and BxPC-3 cells (Fig. 3E-H). Thus, these results suggest that circACTR2 downregulation is essential for sustaining GEM resistance and that circACTR2 overexpression substantially increased the efficacy of GEM by inducing tumor cell apoptosis.

### circACTR2 reverses GEM resistance by acting as miRNA sponge for miR-221-3p

**Fig. 4:**
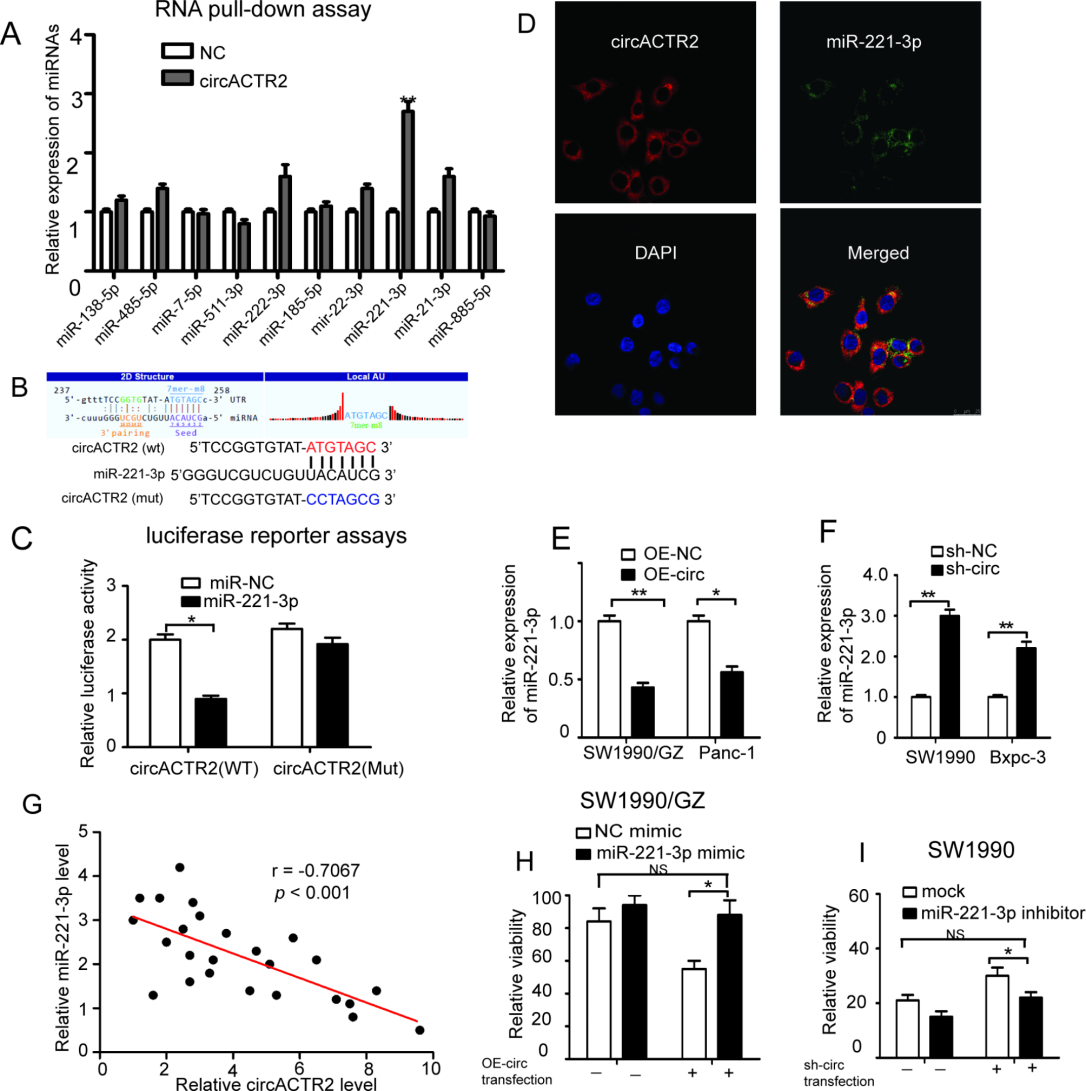
circACTR2 reverse GEM resistance by acting as an miRNA sponge for miR-221-3p **A** qRT-PCR analysis for predicted miRNAs pulled-down by circACTR2. **B** Schematic illustration of wild-type and mutant circACTR2 luciferase reporter vectors. **C** Dual-luciferase reporter assays using the linear form of wild-type and mutant circACTR2 in SW1990 cells transfected with NC or miR-221-3p mimic. **D** FISH images showed the co-localization of circACTR2 labeled with Cy3 (red) and miR-221-3p labeled with FITC (green) in the cytoplasm of PC cells. nuclei were stained with DAPI. Scale bar, 25 μm. **E** and **F** The relative expression levels of miR-221-3p in PC cells stably transfected with OE-circ or sh-circ vector were detected by qRT-PCR. **G** Pearson correlation analysis of circACTR2 and miR-221-3p expression in 25 PC tissues. **H** and **I** Relative cell viability of PC cell transfected with miR-221-3p inhibitor or mimic compared with NC controls under GEM treatment was determined by CCK-8 assay. *P < 0.05; **P < 0.01

Given that circACTR2 localizes to the cytoplasm, we hypothesized that miRNA sponge activity could be a possible mechanism for its functional effects. 59 miRNAs were predicated and listed as possible targets of circACTR2 based on Arraystar’s miRNA target prediction software based on TargetScan & miRanda (Additional file 2: Table S2). Among above miRNAs, we focused on these miRNAs that have been previously found to be involved in tumor chemo-resistance and AKT signal pathway, such as miR-221-3p, miR-185-5p, miR-138-5p, miR-21-3p, miR-222-3p, miR-7-5p, miR-885-5p, miR-485-5p, miR-22-3p and miR-511-3p. RNA pull-down assay were further performed to analyse the 10 candidate miRNAs, which showed a specific enrichment of miR-221-3p as compared to the controls (Fig. 4A). Thus we speculated that circACTR2 may regulated GEM resistance by acting as miRNA sponge for miR-221-3p. According to the predicted miRNA binding sites on circACTR2, luciferase reporter assays showed that miR-221-3p could specifically target the wild-type linear form of circACTR2 resulting in decreased luciferase activity, but not the mutant form (Fig. 4B, C). Consistently, RNA FISH indicated that circACTR and miR-221-3p were co-localized in the cytoplasm (Fig. 4D). Additionally, circACTR2 overexpression led to markedly decrease of miR-221-3p and circACTR2 knockdown could significantly increase the expression of miR-221-3p in PC cells (Fig. 4E, F). Pearson correlation analysis further indicated that the expression levels of circACTR2 were negatively associated with those of the miR-221-3p in PC tissues (Fig. 4G). These results suggested circACTR2 could act as an miRNA sponge for miR-221-3p.

To determine whether circACTR2 overexpression reverse GEM resistance by sponging miR-221-3p, we first transfected miR-221-3p mimic in SW1990/GZ cells, which showed increased GEM resistance, and miR-221-3p mimic could largely attenuates the inhibitory effect of circACTR2 overexpression on GEM resistance in SW1990/GZ cells (Fig. 4H). Conversely, miR-221-3p inhibitor could increase GEM sensitivity in SW1990 cells and abolish the promoting effect of circACTR2 knockdown on GEM resistance in SW1990 cells (Fig. 4I). These data demonstrated miR-221-3p as target for circACTR2 to mediated its regulatory role in the GEM resistance.

### CircACTR2 reverses GEM resistance by inhibiting PI3K/AKT pathway via miR-221-3p/PTEN axis

**Fig. 5:**
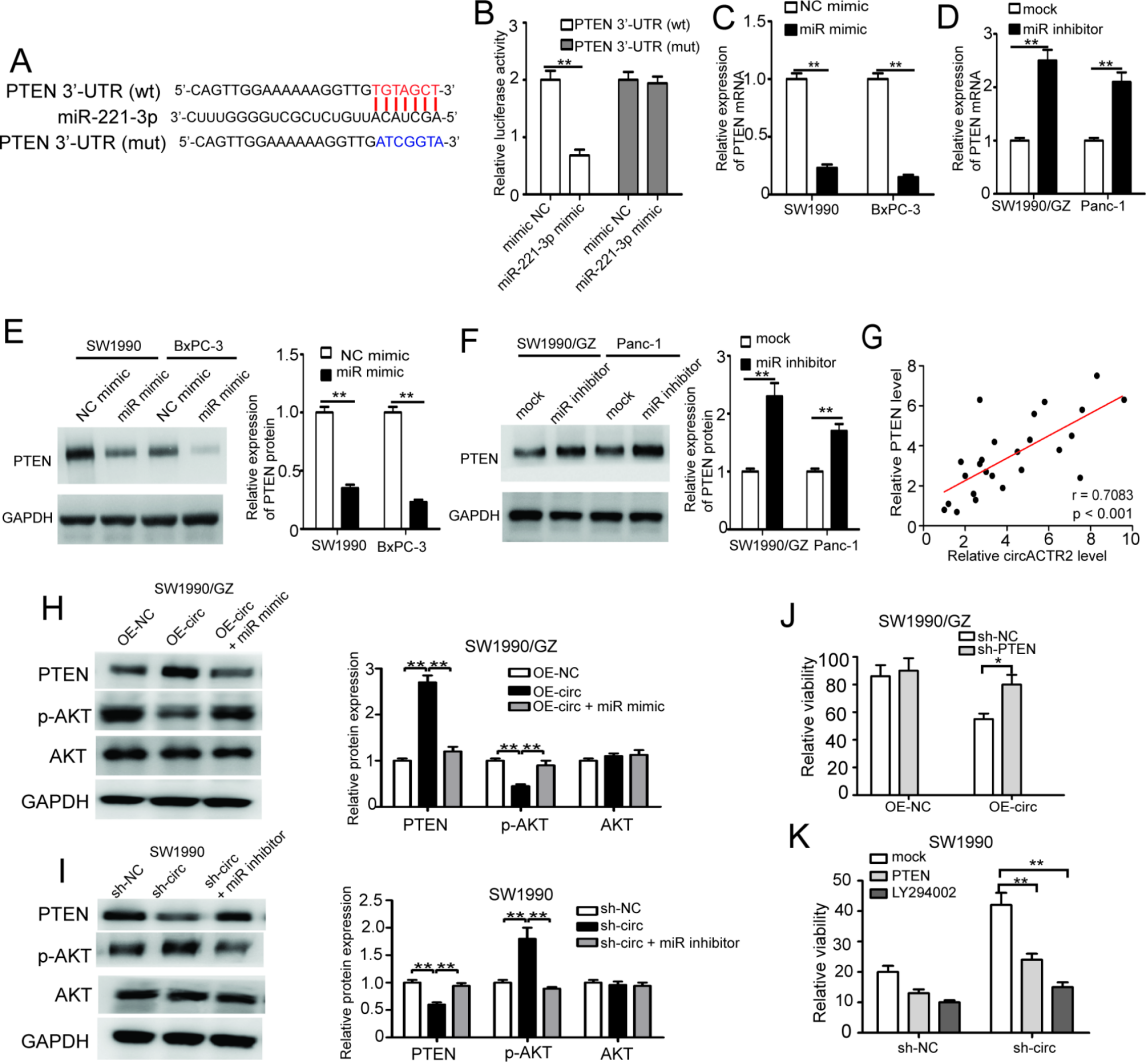
circACTR2 reverse GEM resistance by inhibiting PI3K/AKT pathway via miR-221-3p/PTEN axis **A** Schematic graph illustrated the mutation of potential binding site between miR-221-3p and the 3’-UTR regions of PTEN. **B** The relative luciferase activities of wild type PTEN 3’UTR and its mutant after transfected with miR-221-3p mimic in SW1990 cells. **C** and **D** PETN expression in PC cells after over-expressing and silencing miR-221-3p were detected by qRT-PCR, respectively. **E** and **F** PETN expression in PC cells after over-expressing and silencing miR-221-3p were detected by Western blotting, respectively. **G** Pearson correlation analysis of circACTR2 and PTEN expression in 25 PC tissues. **H** and **I** Western blotting for PTEN, AKT and p-AKT in PC cell lines transfected with indicated vectors, miR mimic or inhibitors. **J** and **K** Relative cell viability of PC cells were determined after treated with sh-PTEN transfection or PTEN OE-vector/LY294002 by CCK-8 assays, respectively. *P < 0.05; **P < 0.01

To elucidate the downstream mechanisms of circACTR2, based on above GO and pathway analysis suggesting circACTR2 might negatively regulate PI3K/AKT signal which is often abnormally activated to promote chemo-resistance of PC (Fig. 1F). What draws our attention is that target gene prediction shows that miR-221-3p contains complementary sequences of PTEN 3’UTR region, a key negative upstream regulator of PI3K/AKT signaling pathway [14]. To valid this, reporter assays demonstrated that miR-221-3p decreased the luciferase activity of the wild-type reporter, while no significant changes were found using mutant reporters (Fig. 5A, B). Consistently, the expression of PTEN mRNA and protein could be regulated by altering miR-221-3p expression with miR-221-3p mimic and inhibitor transfection (Fig. 5C-F). Pearson correlation analysis further indicated that the expression levels of circACTR2 were positively associated with those of the PTEN in PC tissues (Fig. 5G). Furthermore, circACTR2 overexpression inhibited the PI3K/AKT signal pathway shown as remarkable decrease of p-AKT expression, while circACTR2 knockdown led to the opposite results, which could be largely abolished by co-transfected with miRNA-221-3p mimic and inhibitor respectively (Fig. 5H, I).

To further assess the role of PTEN/PI3K/AKT pathway in mediating circACTR2 functions, rescue assays indicated that PTEN knockdown reversed the inhibitory effect of circACTR2 overexpression on GEM resistance in SW1990/GZ cells, while PTEN overexpression or PI3K inhibitor LY294002 abolished the promoting effect of circACTR2 knockdown on GEM resistance in SW1990 cells (Fig. 5J, K). These results together showed that circACTR2 can upregulate PTEN expression by specifically sponging miR-221-3p, thereby inhibit the PI3K/AKT pathway to attenuate GEM resistance.

### circACTR2 overexpression retards PC GEM resistance in vivo

**Fig. 6:**
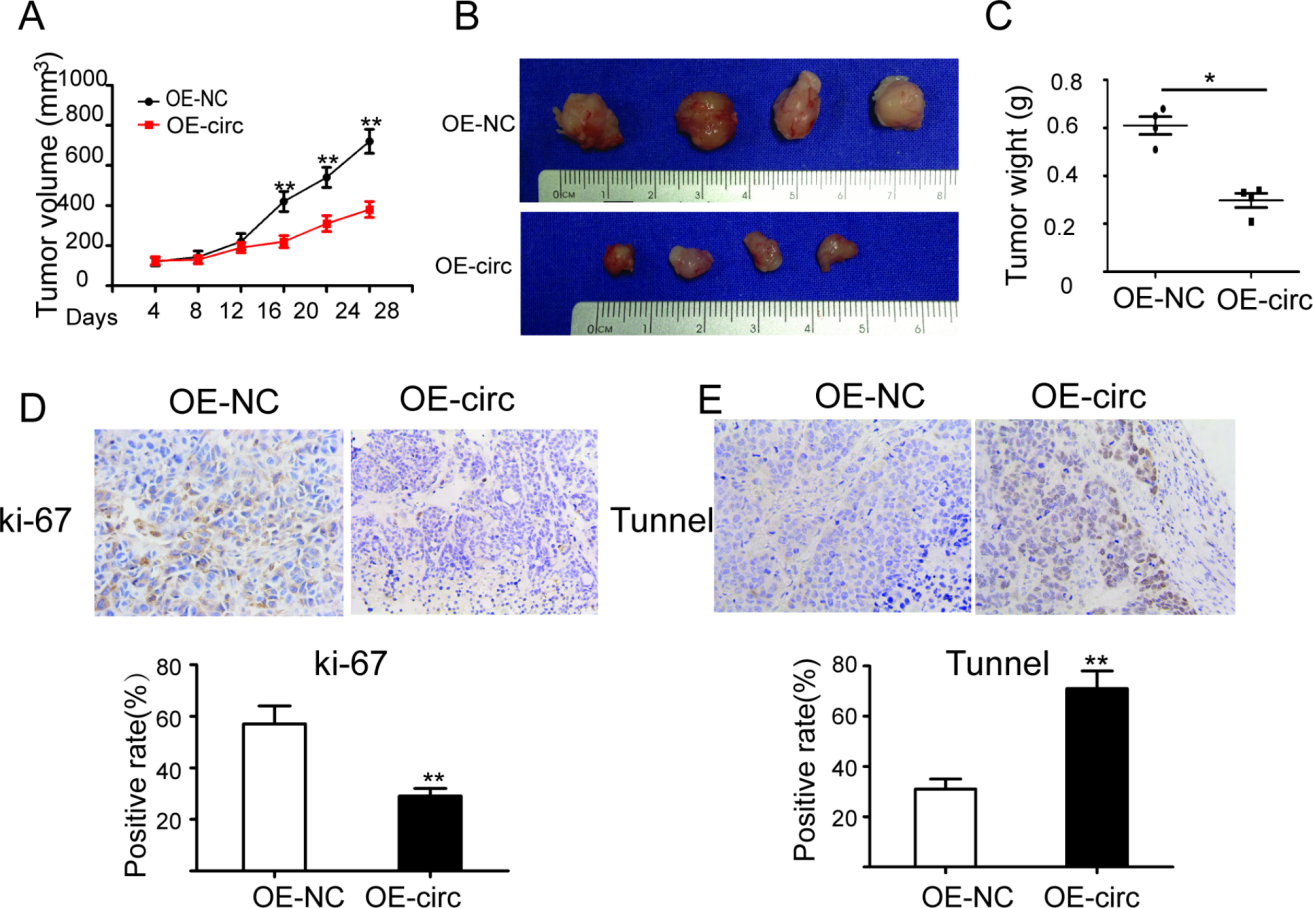
Targeting circACTR2 in vivo retards PC GEM resistance **A** Tumor volume was measured every four days in OE-circ and OE-NC groups after the gemcitabine injections (n = 4). **B** Pictures of tumors and **C** tumors weight in OE-circ and OE-NC groups 4 weeks after the GEM treatment. Immunohistochemical staining of PCNA **D** and tunnel **E** in OE-circ and OE-NC group tumors (×200). Percentage of PCNA-positive and tunnel-positive cells was shown for each group. *P < 0.05; **P < 0.01

To further determine the effects of circACTR2 on GEM resistance in vivo, we subcutaneously injected OE-circ and OE-NC transfected SW1990/GZ cells into nude mice exposed to GEM treatment. The results showed a significant tumor growth inhibition in OE-circ group as compared OE-NC group (Fig. 6A), indeed, the tumor weight was significantly less in the OE-circ group than that in OE-NC group (Fig. 6B, C). Similar to in vitro results, IHC assay showed that the percentage of PCNA-positive cells in OE-circ group was lower than OE-NC group (Fig. 6D). Conversely, the percentage of PTEN-positive and tunnel-positive cells in OE-circ group was significantly higher than OE-NC group (Fig. 6E). These in vivo findings supported that circACTR2 has potential as a novel therapeutic target for GEM resistance in PC.

## Discussion

PC has a high degree of malignancy without obvious symptoms at the early stage of disease, thus most patients are diagnosed at the late stage when chemotherapy has become the main treatment. GEM is still the first-line drug for patients with advanced unresectable PC. Although most patients have initial response to treatment, chemotherapy resistance will gradually appear, which is the main reason for treatment failure [15]. The exploration of the mechanism underlying GEM resistance has been continuing, including the decrease of drug uptake efficiency, the expression of drug efflux transporters, the increase of enzymatic drug inactivation, the damage of cell apoptosis, the activation of epithelial mesenchymal transformation, and the stem pathway [16]. In recent years, non-coding RNA, especially circRNA, as a new epigenetic regulatory mechanism, has been found to play an important role in the pathogenesis of cancer, and can regulate the sensitivity of cancer cells to different chemotherapy drugs [17]. For example, circ_0013587 reverses erlotinib resistance in PC cells through regulating the miR-1227/E-Cadherin pathway [18]. Another study reported that circHIPK3 can promote GEM resistance in PC cells by sponging miR-330-5p and targets rassf1 [19]. However, the role of circRNA in acquired drug resistance of PC is still largely unclear. Thus, we have previously performed high-throughput microarray analysis in acquired GEM-resistant cells, showing several circRNAs are significantly dysregulated [11]. Among them, our study showed that circACTR2 is negatively associated with aggressive cell phenotype and poor prognosis in PC patients. A previous study showed that circACTR2 knockdown significantly decreased high glucose-induced pyroptosis, inflammation and fibrosis in proximal tubular cells [20]. A recent study demonstrated that circACTR2 activated macrophage inflammation, and stimulated macrophage-induced EMT and fibrosis of renal tubular epithelial cells [21].

In our study, functional analysis based on microarray profile of acquired GEM-resistant PC cells suggests that circACTR2 is related to multiple drug resistance related biological processes, which lead us to speculate that circACTR2 is involved in GEM resistance. To validate that, the gain/loss of function experiments showed that circACTR2 could negatively regulate the sensitivity of PC cells to GEM. Induction of cell apoptosis is one of the main mechanisms of GEM. As a nucleoside analogue, GEM affects cell cycle by inducing cell cycle arrest in S phase when used at low/moderate doses and exerts its cytotoxicity on cells in S phase [22, 23]. Increased S phase ratio of cell cycle by circACTR2 overexpression, indicating that PC cells were blocked in S phase, might be responsible for significant increase in PC cell apoptosis at relatively low doses of GEM, suggesting the potential of circACTR2 as a chemosensitizer of GEM.

CircRNAs have been suggested to function as miRNA sponges to regulate the expression of downstream target genes with following characteristics: (1) derived from protein encoding exons; (2) predominantly located in cytoplasm [24]. Given that circACTR2 derives from the 5th and 6th exons of ACTR2 gene and mainly distributes in the cytoplasm, thus circACTR2 may act as miRNA sponge. circRNA requires specific binding sites for multiple miRNAs to conduct diverse biological roles. Numerous studies reported that circACTR2 could serve as sponge for several miRNAs, such as miR-561 and miR-205-5p [21, 25]. Our study firstly demonstrated that miR-221-3p was a direct target of circACTR2. Notably, miR-221-3p has been reported to act as a tumor promoter in multiple types of cancers, involved in tumor invasion, metastasis, proliferation and chemotherapy resistance by regulating the expression of target genes such as VASH1, PARP1, RB1 [26–28]. Recent studies have demonstrated that miR-221-3p is upregulated and predicts poor prognosis in PC [29, 30]. However, the role of miR-221-3p in GEM resistance in PC is unclear. Indeed, our results suggested that miR-221-3p could negatively regulate sensitivity of PC cells to GEM, and circACTR2 sensitized PC cells to GEM in a miR-221-3p dependent manner. To elucidate the downstream mechanisms of circACTR2, pathway analysis suggested circACTR2 negatively regulate PI3K/AKT signal pathway, which is often abnormally activated to promote chemoresistance of cancers [31]. Bioinformatics prediction further suggested that miR-221-3p may directly target PTEN, which is usually inactivated in a variety of human cancers including PC, results in the activation of the PI3K/Akt pathway [32, 33] Activated Akt can promote cell proliferation, invasion and angiogenesis, but inhibit cell apoptosis, through catalyzing phosphorylation of a series of effectors [34, 35]. Our study confirmed that miR-221-3p could sponge PTEN and downregulate its expression, thereby activate the PI3K/AKT signal pathway, consistent with previous studies [36, 37].

**Fig. 7:**
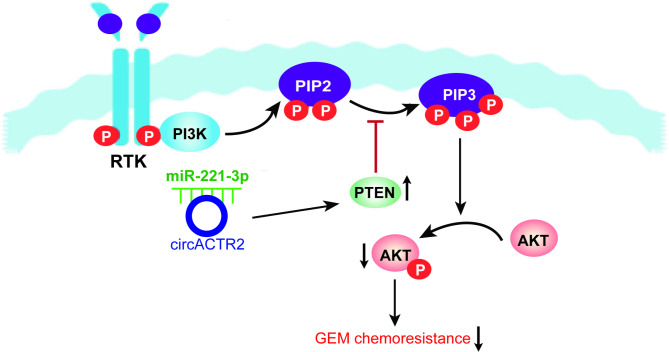
Proposition of a model in which circACTR2 acts as a sponge for miR-221-3p to reduce PC GEM resistance by regulating the PTEN/PI3K/AKT signaling pathway

In summary, circACTR2 was significantly downregulated in GEM-resistant PC cells, contributing to PC GEM resistance. circACTR2 overexpression reversed the chemoresistance of PC cells to GEM through inhibiting PI3K/AKT signaling pathway by sponging miR-221-3p and upregulating PTEN expression (Fig. 7), which might provide an essential hint for circACTR2 as therapeutic target to overcome PC cheomresistance.

## Electronic supplementary material

Below is the link to the electronic supplementary material.

Supplementary Material 1: Table S1. List of primers used for qRT-PCR in this study

Supplementary Material 2: Table S2. List of predicted miRNA targets of circACTR2 based on TargetScan & miRanda in this study

## Data Availability

All the data obtained in current study were available from the corresponding authors on reasonable request.

## Abbreviations

GEM: gemcitabine
PC: pancreatic cancer
DMEM: Dulbecco’s modifed eagle’s medium
circRNA: Circular RNA
TMA: Tissue microarrays
PTEN: Phosphatase and tensin homolog
NF-κB: Nuclear factor κB
FISH: Fluorescence in situ hybridization
IHC: Immunohistochemistry
shRNA: Short hairpin RNA
NC: Negative control
OE: Overexpression
PBS: Phosphate bufer saline
ceRNA: Competing endogenous RNA
miRNA: MicroRNA
