# Supplementary material for: circACTR2 attenuates gemcitabine chemoresiatance in pancreatic cancer through PTEN mediated PI3K/AKT signaling pathway
Source: Biol Direct. 2023 Mar 30;18:14. doi: 10.1186/s13062-023-00368-8 (PMC10061898; doi:10.1186/s13062-023-00368-8)
Supplement: Supplementary file 1 — Supplementary Material 1: Table S1. List of primers used for qRT-PCR in this study [file 13062_2023_368_MOESM1_ESM.doc]

**Table. S1** List for primers used for qRT-PCR.

| Primer sequence |  |  |
| --- | --- | --- |
| circACTR2 | Forward | GTATCCTGGCCTGCCATC |
|  | Reverse | TTGCCTCATCACCAACCA |
| ACTR2 | Forward | GTAGCCATCCAGGCAGTTCT |
|  | Reverse | AAGGCGTATCCTCGCAACAG |
| miR-221-3p | Forward | GCGAAAGTGCTGCGACATTT |
|  | Reverse | CGCGAGCTACATTGTCTGCTG |
| PTEN | Forward | TGGATTCGACTTAGACTTGACCT |
|  | Reverse | GGTGGGTTATGGTCTTCAAAAGG |
| U6 | Forward | CTCGCTTCGGCAGCACA |
|  | Reverse | AACGCTTCACGAATTTGCGT |
| GAPDH | Forward | CCACATCGCTCAGACACCAT |
|  | Reverse | ACCAGGCGCCCAATACG |
